# Validation of a hypomorphic variant in *CDK13* as the cause of CHDFIDD with autosomal recessive inheritance through determination of an episignature

**DOI:** 10.1186/s13148-024-01807-7

**Published:** 2025-01-13

**Authors:** Jan Fischer, Mariëlle Alders, Marcel M. A. M. Mannens, David Genevieve, Karl Hackmann, Evelin Schröck, Bekim Sadikovic, Joseph Porrmann

**Affiliations:** 1 Faculty of Medicine of TUD Dresden University of Technology, Institute for Clinical Genetics, University Hospital Carl Gustav Carus at TUD Dresden University of Technology, Dresden, Germany; 2 Department of Human Genetics, Amsterdam Reproduction and Development Research Institute, Amsterdam UMC, University of Amsterdam, Meibergdreef 9, 1105 AZ Amsterdam, The Netherlands; 3 Genetic Department, Reference Center for Abnormal Development and Malformative Syndrome, Montpellier University, ERN ITHACA, CHU Montpellier, Inserm Unit 1183, Montpellier, France; 4 Max Planck Institute of Molecular Cell Biology and Genetics, Dresden, Germany; 5 Verspeeten Clinical Genome Centre, London Health Sciences Centre, London, ON N6A 5W9 Canada; 6 Department of Pathology and Laboratory Medicine, Western University, London, ON N6A 3K7 Canada; 7 Faculty of Medicine of TUD Dresden University of Technology, University Centre for Rare Diseases, Dresden, Germany

**Keywords:** CDK13, CHDFIDD, Developmental delay, Episignature

## Abstract

Autosomal dominant *CDK13*-related disease is characterized by congenital heart defects, dysmorphic facial features, and intellectual developmental disorder (CHDFIDD). Heterozygous pathogenic variants, particularly missense variants in the kinase domain, have previously been described as disease causing. Using the determination of a methylation pattern and comparison with an established episignature, we reveal the first hypomorphic variant in the kinase domain of *CDK13,* leading to a never before described autosomal recessive form of CHDFIDD in a boy with characteristic features. This highlights the utility of episignatures in variant interpretation, as well as a potential novel diagnostic approach in unsolved cases or for disease prognosis.

## Introduction

*CDK13*-related disorder is an autosomal dominant disease whose acronym, CHDFIDD, is derived from its characteristic features, namely congenital heart defects, dysmorphic facial features, and intellectual developmental delay (MIM #617,360) [1, 2]. CHDFIDD is the result of heterozygous pathogenic variants in the cyclin dependent kinase 13 (*CDK13*) gene, which encodes an important transcriptional regulator. It forms functional complexes with Cyclin K (CCNK) and phosphorylates serine residues (specifically Ser2) in the C-terminal domain of RNA polymerase II to alter its transcription of genes, particularly in growth signaling pathways [3]. *CDK13* has a close paralog, *CDK12*, which also complexes with Cyclin K with comparable, yet not identical, effects on gene transcription [4].

**Fig. 1:**
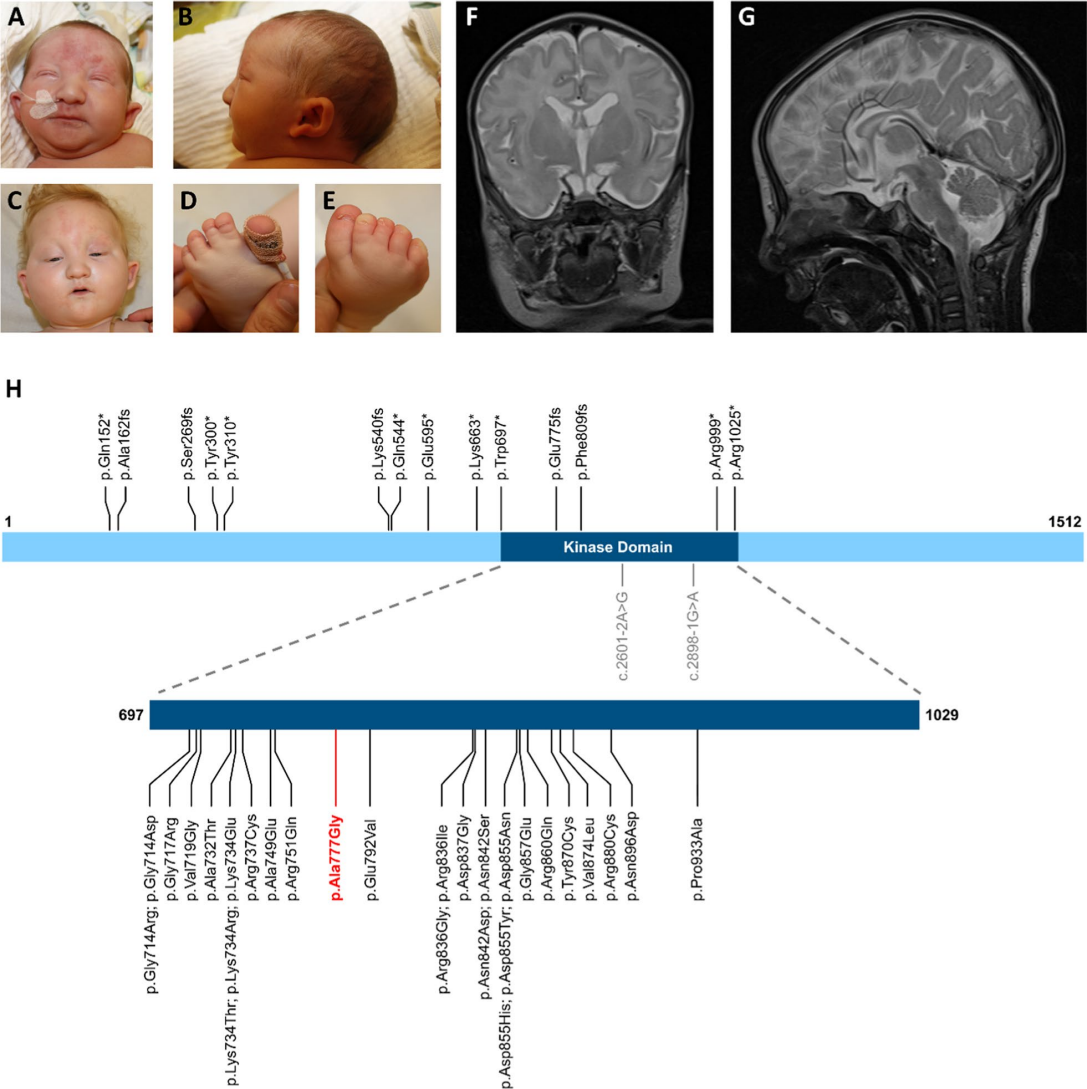
**A**–**B** Facial images at 5 days. **C** Facial image at 10 months. **D**–**E** Bilateral syndactyly of toes 2 and 3. **F**–**G** Brain MR image at 14 months of age showing corpus callosum hypoplasia and cerebellar hypoplasia. **H** Schematic of the CDK13 protein showing published loss-of-function and splice variants, as well as missense variants in the kinase domain associated with CHDFIDD. The hypomorphic variant p.Ala777Gly is highlighted in red

**Fig. 2:**
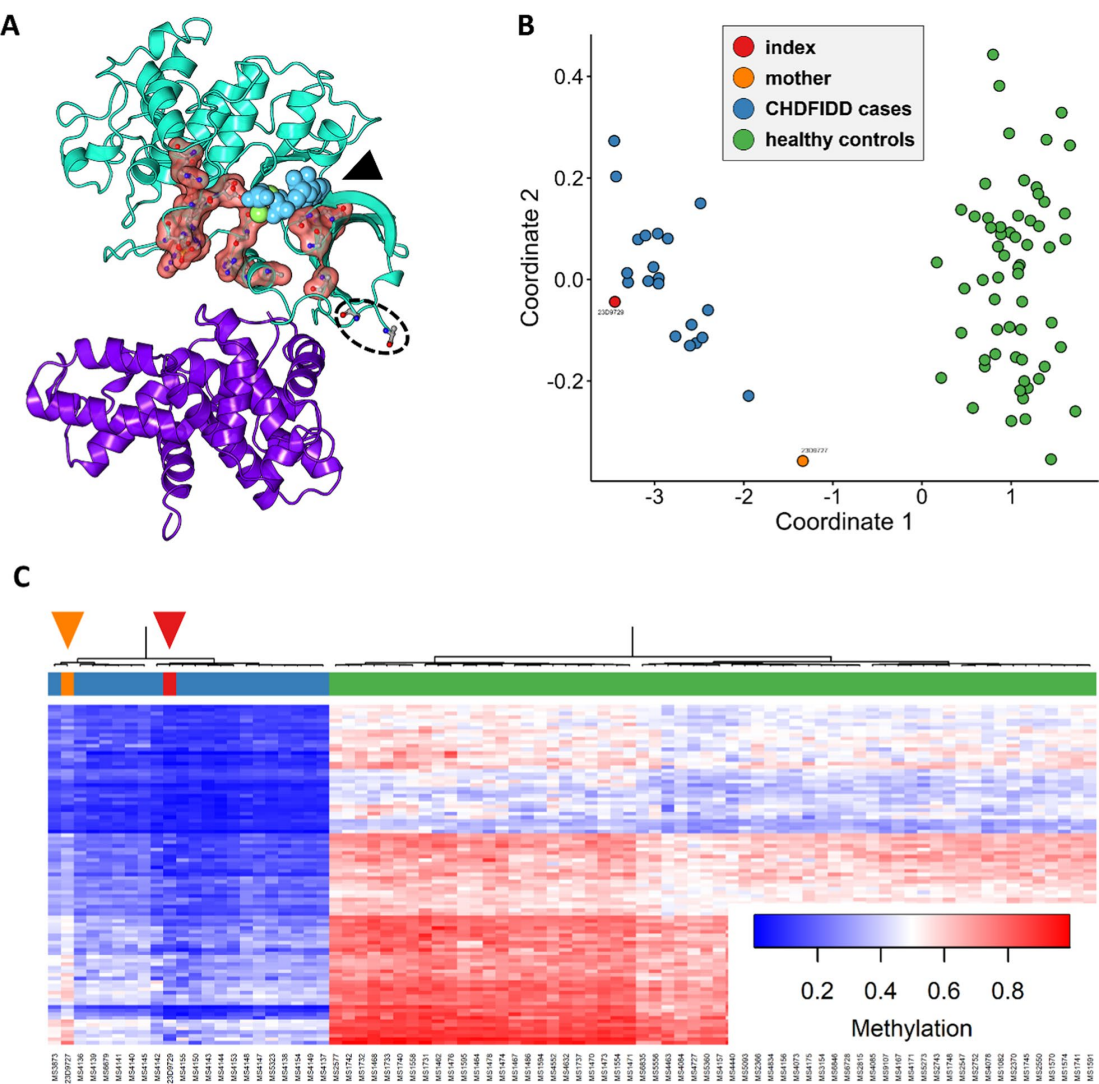
**A** Rendering of the crystal protein structure of the kinase domain of CDK13 (turquoise) and Cyclin K (purple) (RCSB-PDB). Localization of the ATP-binding pocket is shown (black arrow). Amino acids involved in known pathogenic missense variants are highlighted in red. The last amino acids resolved in the crystal structure of the β4-β5 loop containing p.Ala777 are encircled (p.Asp776 & Lys781). **B** Principal component analysis of the episignatures of previously published CHDFIDD patients (blue) and controls (green) [5] as well as our patient with homozygous p.Ala777Gly (red) and our patient’s mother with heterozygous p.Ala777Gly (orange). **C** Hierarchical clustering heatmap of the aforementioned groups

While some disease-causing nonsense, frameshift, and splice variants have been described in *CDK13*, the majority of pathogenic variants are amino acid substitutions that are only found in the kinase domain (Fig. 1H). Within the domain, pathogenic missense variants cluster particularly around the ATP-binding pocket (Fig. 2A). This disrupts the catalytic activity of the enzyme but likely leaves the binding of CDK13 to Cyclin K undisturbed, thereby creating a dominant negative effect [2].

Importantly, CDK13 also affects DNA methylation and individuals with CHDFIDD exhibit general hypomethylation of CpG sites throughout the genome [3, 5]. A genome-wide methylation screen (EpiSign™ DNA methylation analysis) has previously been used to compare a CHDFIDD patient cohort to healthy controls in order to establish a characteristic episignature (DNA methylation classifier) for CHDFIDD [5]. This *CDK13*-specific episignature consists of 140 differentially methylated (hypomethylated) probes. Methylation patterns for undiagnosed patients can now be compared with a disease-specific episignature. Probability estimates, termed methylation variant pathogenicity (MVP) scores, can further indicate confidence in the association of an individual’s methylation pattern with that of a particular disease cohort. An MVP score of > 0.5 has been suggested to be indicative of *CDK13*-associated disease [5].

Overall, this episignature is part of a growing number of DNA methylation classifiers that have been established for a number of heritable illnesses [6]. Each episignature is made up of disease-specific combinations of differentially methylated CpG sites and is established from patient cohorts of varying sizes [6].

### Case presentation

Photographs and brain MR images are shown in Fig. 1. The boy was last seen in the genetics outpatient clinic in Dresden, Germany, at the age of 11 months. The pregnancy was reported as initially unremarkable. Late preterm birth occurred at 36^+2^ weeks of gestation after spontaneous onset of labor. Cardiotocographic abnormalities were present during labor. APGAR scores of 5, 6, and 9 were recorded at 1 min, 5 min, and 10 min after birth. Neonatal acidosis was present with a cord arterial pH of 7.09. Owing to acute respiratory distress, noninvasive ventilation with continuous positive airway pressure (CPAP) was commenced. He had a birth weight of 2051 g (2nd percentile, -1.98 SD), a length of 44 cm (2nd percentile, -2.14 SD), and a head circumference of 33 cm (27th percentile, -0.62 SD). A cephalohematoma with subsequent anemia was also noted, later necessitating a blood transfusion. Echocardiography revealed an atrial septal defect (secundum type) and mild pulmonary valve stenosis. A brain MRI revealed corpus callosum hypoplasia and cerebellar hypoplasia. A horseshoe kidney, cryptorchidism, and an inguinal hernia were further noted. No hearing or visual impairments were reported. Due to feeding difficulties in the 1st year of life, a percutaneous endoscopic gastrostomy (PEG) was placed. At the age of 11 months, secondary microcephaly was noted (OFC: 44 cm, 2nd percentile, -2.14 SD; weight: 8.6 kg, 12th percentile, -1.17 SD; length: 75.5 cm, 47th percentile, -0.07 SD). Moderate-to-severe global developmental delay was present. Speech development was assessed to be on the 4-month-old. Monosyllabic, but not polysyllabic, vocalization was observed. Hand and gross motor skills were deemed comparable to those of 6- to 7-month-old individuals with the use of a palmar grasp and the ability to roll over, yet without any further efforts at locomotion.

The boy had pronounced facial dysmorphisms with hypertelorism, narrow palpebral fissures, sparse eyebrows, protruding, cupped ears with a hypoplastic crus helix and a narrow mouth with full cheeks. Bilateral clubfoot with bilateral syndactyly of toes 2 and 3 was also noted. Face2Gene showed a high index of similarity to CHDFIDD but also notable overlaps with Ohdo syndrome (MIM #300,895) and STAR syndrome (MIM #300,707).

His consanguineous parents both had learning difficulties without further known signs of CHDFIDD and no facial dysmorphisms in the mother. Face2Gene showed no relevant similarities to known syndromic diseases for the mother. The boy’s paternal half-brother had developmental delay with confirmation of a pathogenic deletion in 22q13 causing Phelan–McDermid syndrome. This, along with other copy number variants, had previously been excluded through Array CGH analysis in our index case.

### Molecular studies

Exome-wide sequencing was carried out for the index case on DNA extracted from blood-derived lymphocytes. Exome capture was performed via the Twist Human Core Exome Kit according to the manufacturer’s specifications. 150 nt paired-end sequencing was performed on a NovaSeq 6000 Sequencer (Illumina). Alignment (mapping to GRCh37/hg19), variant identification (incl. single-nucleotide variants and copy number variants), variant annotation, and filtering were carried out using the Varvis genomics platform (Limbus Medical Technologies).

The homozygous missense variant NM_003718.5:c.2330C > G, p.(Ala777Gly), which lies within the kinase domain of *CDK13*, was detected. It was found within a run of homozygosity of approximately 45 Mb on chromosome 7. It has never been previously reported in any population database (incl. gnomAD v4.1.0) or in the literature. AlphaMissense predicted a deleterious effect, whereas Revel provided no clear prediction (uncertain 0.33). The alanine at this position has been highly conserved throughout evolution, and it, as well as the surrounding amino acids, has remained unchanged in all vertebrates. No further variants of interest were detected. Segregation analysis confirmed that the mother is a heterozygous carrier of the variant. The father was not available for genetic testing.

Methylation analysis with the EpiSign™ assay was carried out for the boy and his mother using DNA extracted from blood according to previously published protocols at Amsterdam University Medical Center [5, 7]. Briefly, an Infinium methylation EPIC Bead Chip array was performed with the data being imported into R (version 4.2.3) for further processing and comparison to the previously established CHDFIDD-specific episignature [7]. The controls and CHDFIDD patients (with pathogenic *CDK13* missense variants) used for comparison were largely part of the previously published study by Rouxel et al. (2022) [5]. Upon comparison with the CHDFIDD-specific episignature, an MVP score of 0.98 was determined for the boy. The heterozygous mother had an MVP score of 0.45.

## Discussion

We report the novel missense variant p.Ala777Gly in *CDK13*. It is homozygous in the index patient and heterozygous in his mother. The variant was initially classified according to the guidelines of the American College of Medical Genetics and Genomics and the Association for Molecular Pathology (ACMG/AMP) as a variant of unknown significance, with the criteria PM1 (located in a critical functional domain) and PM2_SUP (absent in control cohorts) being fulfilled. A genome-wide methylation screen revealed that the index displayed a methylation pattern that closely mirrors the previously established CHDFIDD-specific episignature (Fig. 2B) [5]. Multidimensional scaling indicates that the index case clusters well among known CHDFIDD cases. Furthermore, an MVP score of 0.98 indicates a very high degree of confidence in the association with *CDK13*-associated disease. A second DNA methylation analysis was carried out for the heterozygous mother. She did not exhibit a CHDFIDD-specific (hypo)methylation pattern; yet, her MVP score of 0.45 only fell marginally below the cutoff of 0.5 for *CDK13*-associated disease. Importantly, she presented a much greater degree of hypomethylation than healthy controls. Multidimensional scaling highlights that she neither clusters with known CHDFIDD cases nor with healthy controls (Fig. 2B).

Taken together, these data suggest that p.Ala777Gly is the first known hypomorphic variant in *CDK13* that leads to autosomal recessive CHDFIDD. Although no consensus exists on how episignatures can be incorporated in the evaluation of genetic variants, in light of the methylation findings and previously published recommendations, we applied the ACMG/AMP criteria PS3_MOD (functional data) [8]. Moreover, under the assumption that an autosomal recessive inheritance pattern can be confirmed in other cases, PM3_SUP (homozygous variant in recessive disease) could also be applied. This, in conjunction with the aforementioned criteria as well as with the overlap in phenotype, would yield an evaluation of p.Ala777Gly as a likely pathogenic variant. When present in a heterozygous state, the hypomorphic variant p.Ala777Gly may be associated with mild nonsyndromic intellectual disability. The heterozygous mother displayed learning difficulties without any of the other signs of CHDFIDD, most notably no facial dysmorphisms. However, polygenic features and environmental factors cannot be excluded as causative components.

The variant p.Ala777Gly lies within the kinase domain. Although the crystal structure has not been fully resolved for the region of CDK13 containing this amino acid (and the three subsequent amino acids), it is likely to lie far outside the ATP-binding pocket (RCSB PDB: 5EFQ) (Fig. 2A). Comparisons with the structurally very similar protein CDK12 suggest that it lies within a β4-β5 loop of the N-terminal lobe of the domain and is located at the protein interface to Cyclin K [4]. Unlike other pathogenic missense variants in the kinase domain, p.Ala777Gly may therefore not lead to conformational changes of the ATP-binding pocket but rather results in reduced or unstable CDK13-Cyclin K binding. A smaller reduction in overall catalytic activity may be observed, especially as no dominant negative effect or haploinsufficiency would be present. Additional functional studies including Cdk13 substrate binding and substrate phosphorylation assays may be considered to further validate this effect [3].

Overall, this case highlights the ability to improve variant interpretation via disease-specific episignatures. Methylation patterns can provide important functional insights for individual variants and can thereby be used as indicators of variant pathogenicity. This allows for increased diagnostic accuracy and reduces the chance that important genetic diagnoses are missed or underreported due to uncertainties in variant interpretation. Suggestions have been made regarding how episignature information can be used to assign certain pathogenicity criteria; yet, clinicians and those evaluating sequencing data would benefit from greater formalization.

Face2Gene also showed significant overlap of facial dysmorphic features between our CHDFIDD patient with STAR syndrome as well as Ohdo syndrome (Type Maat-Kievit-Brunner). Notably, no variants of interest were found in the associated genes. STAR syndrome is caused by pathogenic variants in Cyclin Q (CCNQ) [9]. CCNQ forms complexes with cyclin dependent kinase 10 (CDK10) and phosphorylates some of the same serine residues of RNA polymerase II [9]. Similarly, X-linked Ohdo syndrome is also associated with altered RNA polymerase II-based transcriptional regulation [10]. Disease-causing variants are found in *MED12*, which forms part of a kinase module (together with Cyclin C, MED13, and CDK8) that belongs to the larger Mediator complex. The Mediator complex, in turn, affects transcription initiation by RNA polymerase II. As CDK13 also affects transcription through RNA polymerase II, the similarities between CHDFIDD, STAR syndrome and Ohdo syndrome may result from alterations in common cellular pathways, leading to comparable disease manifestations and facial features. Unfortunately, no episignature could thus far be determined for Ohdo syndrome (possibly owing to a small sample size) [6] or STAR syndrome (no published efforts for episignature determination). We therefore emphasize the need for further collaborative efforts for methylation analysis in known cases of these diseases, as well as other disease entities that are associated with alterations in RNA polymerase II function. This can provide valuable insight into whether RNA polymerase II-associated diseases share common methylation patterns while still being epigenetically distinguishable from one another and thereby direct the focus during the search for disease-causing variants.

Finally, studies of episignatures in other diseases have suggested that the degree of methylation deviation in affected individuals may correlate with overall disease severity or age of onset [11]. The variant p.Ala777Gly is now able to provide similar insights for CDK13. The mildly affected heterozygous mother exhibits an intermediate deviation of methylation, whereas the homozygous index showed a methylation deviation that matched that of other individuals with CHDFIDD. Thus, the extent of methylation alteration can potentially give insights into disease prognosis. This may be particularly valuable for young infants and children to provide early insight into disease severity to treating clinicians and allow for timely introduction of necessary support measures and suitable determination of follow-up intervals.

Taken together, the presented case strongly highlights the utility of episignatures in the clinical setting. This not only allows for improved variant interpretation for genetic alterations of unknown significance but can also enable the diagnosis of novel disease entities or heredity. This is showcased by the validation of the novel hypomorphic *CDK13* variant described herein, which led to the first ever published case of autosomal recessive CHDFIDD. Additionally, episignatures may also have potential applications in disease prognosis and mutation search strategies for unsolved cases, making them an important tool in the diagnostic arsenal of modern clinical genetics.

## Data availability statement

The data supporting the current study have not been deposited in a public repository to protect individual confidentiality but are available from the corresponding author upon request.
